# Cognitive-behavioral therapy focused on inhibitory learning, for adults with avoidant/restrictive food intake disorder (ARFID): Study protocol of a prospective study

**DOI:** 10.1371/journal.pone.0354232

**Published:** 2026-07-31

**Authors:** Malou Masereel, Sebastian Cardona Cano, Trung Dung Tran, Margo de Jonge, Eric Dumont, Annemarie A. van Elburg, Sandra Mulkens

**Affiliations:** 1 Department of Clinical Psychological Science, Maastricht University, Maastricht, the Netherlands; 2 Parnassia Psychiatric Institute, The Hague, the Netherlands; 3 Department of Methodology and Statistics, Maastricht University, Maastricht, the Netherlands; 4 Novarum Center for Eating Disorders & Obesity, Amstelveen, the Netherlands; 5 Department of Research and Development, SeysCentra, Malden, the Netherlands; 6 Department of Clinical Psychology, Utrecht University, Utrecht, the Netherlands; 7 Co-Eur Eating Disorders, Vught, the Netherlands; PLOS: Public Library of Science, UNITED KINGDOM OF GREAT BRITAIN AND NORTHERN IRELAND

## Abstract

**Objective:**

Currently, there are no evidence-based treatments available for adults with avoidant/restrictive food intake disorder (ARFID). The present paper describes the design of a study evaluating a cognitive-behavioral therapy (CBT) protocol for adults with ARFID, with a focus on exposure using inhibitory learning principles.

**Method:**

This multicenter study uses a prospective, uncontrolled, repeated-measures design. A total of 120 adults (aged ≥ 18 years) with ARFID will be included via 11 participating treatment centers throughout the Netherlands. Participants will receive a manualized treatment of 25 weekly sessions. Assessments will be conducted at baseline, post-treatment, and at 1- and 12-month follow-ups. The primary outcome is ARFID severity, measured by the Pica, ARFID, and Rumination Disorder Interview (PARDI). Session-level measures of fear and expectancies will be collected throughout the treatment to explore potential mechanisms of change. Treatment effects will be examined across ARFID profiles and comorbid disorders.

**Discussion:**

This is the first large-scale study to investigate an outpatient CBT program for adults with ARFID. By examining outcomes across ARFID profiles and exploring underlying mechanisms of change, the findings are expected to inform treatment refinement and provide a foundation for future controlled trials. The study has been preregistered in AsPredicted (#212,212) and the trial was registered in the Overview of Medical Research in the Netherlands (NL-OMON6121).

## Introduction

In 2013, avoidant/restrictive food intake disorder (ARFID) was introduced to the chapter of feeding and eating disorders in the Diagnostic and Statistical Manual of Mental Disorders, fifth edition (DSM-5) [1]. ARFID refers to a feeding and eating disorder which is characterized by insufficient and/or highly selective food intake, with significant consequences for physical and/or psychosocial wellbeing. Unlike other eating disorders, ARFID is not driven by concerns about body weight or shape. The DSM-5-TR [2] describes three non-mutually exclusive profiles regarding the drivers for avoidance and/or restriction: (1) avoidance based on the sensory characteristics of food (such as taste, smell, color, and texture); (2) a lack of interest in food or eating; and (3) avoidance based on concern over aversive consequences of eating (e.g., choking, gastrointestinal discomfort, or vomiting).

Common psychiatric comorbidities with ARFID include anxiety disorders, neurodevelopmental disorders such as autism spectrum disorder (ASD), attention-deficit/hyperactivity disorder (ADHD), and intellectual disability (ID), as well as mood disorders [3–5]. Somatic comorbidities such as gastrointestinal (GI) symptoms and disorders, metabolic diseases and syndromic disorders are also commonly observed [3,6–8].

ARFID can occur across the lifespan, with prevalence estimates in the general adult population ranging from 0.3% to 4.8% [9–13]. A recent quality-effects meta-analysis that included both clinical and non-clinical samples, found comparable prevalence rates among children (4.73%) and adults (5.9%) [14]. Despite this, evidence-based treatments for adults with ARFID are lacking. Most knowledge about treatment effectiveness to date comes from research in children, most of it conducted before ARFID was introduced as a formal diagnosis in the DSM-5 [1].

For young children with ARFID, behavioral interventions are commonly used, often utilizing strategies like shaping, escape extinction, reinforcement, and systematic desensitization [15–18]. However, these treatment programs are less suitable for adolescents and adults because of the child-focused approach and the lack of consideration for cognitive processes. For this age group, cognitive behavioral therapy (CBT) might be more appropriate. CBT is a well-established and evidence-based treatment for other eating disorders such as bulimia nervosa and binge eating disorder [19–22]. Moreover, CBT is currently used to treat ARFID in clinical practice, and a small number of case studies and case series have examined its application in adolescents (aged 10 years and older) and adults [15].

Dumont and colleagues [23] developed a four-week CBT group intervention for adolescents within an intensive day care, tertiary treatment setting. The treatment protocol includes several CBT components, such as psychoeducation, relaxation techniques, and cognitive restructuring. However, its primary focus is on exposure therapy, with an emphasis on expectancy violation, consistent with the inhibitory learning approach [24]. The intervention does not tailor its approach based on the ARFID profiles. Instead, the authors argue that expectancy violation can be possible across all ARFID presentations, even those not primarily driven by anxiety, by targeting learned associations such as disgust or aversive physiological responses. They evaluated the treatment in a clinical case series with 11 adolescents aged 10–18 years and found that 3 months post-treatment, 10 out of 11 patients were in remission and achieved a healthy body weight and nutritional intake.

Although clinical reports indicate the use of CBT for adults with ARFID, formal evaluation of its effectiveness in this population is lacking. To our knowledge, only one study has investigated a CBT protocol for adults with ARFID. Thomas and Eddy [25] designed a protocol, ‘CBT-AR’, for children from age 10, adolescents and adults with ARFID. CBT-AR consists of 4 stages, spread out over 20–30 sessions. Unlike the protocol of Dumont and colleagues [23], CBT-AR tailors treatment based on the ARFID profile, weight status, and age. Thomas et al. [26] investigated the protocol in 14 adults aged 18–55 years. They found that ARFID severity decreased significantly from pre- to post-treatment and that 47% of the patients no longer met criteria of ARFID immediately after the treatment.

While both the CBT-AR protocol [26] and the day-treatment protocol by Dumont et al. [23] have shown promising results in terms of feasibility and effectiveness, studies with larger sample sizes and longer follow-up periods are still needed. Moreover, the treatment protocol developed by Dumont and colleagues has not yet been evaluated in an adult population, nor has its applicability been examined in a less intensive, secondary care setting. Therefore, the present study aims to evaluate an adapted version of this protocol, modified for adult patients and delivered in an outpatient, secondary care, individual format.

The present study aims to evaluate a 25-session outpatient CBT protocol for adults with ARFID using a multicenter, prospective, one-group design. We will assess changes in ARFID severity from pre- to post-treatment, as well as the stability of these changes during a follow-up period of one year (research question 1). Additionally, we will examine whether treatment effects differ across the three ARFID profiles (research question 2) and whether potential comorbidity affects the outcomes (research question 3). Finally, we will investigate whether underlying mechanisms of change are better explained by habituation or expectancy learning (research question 4).

## Method

### Participants

We aim to include 120 patients across 11 specialized treatment centers for eating disorders throughout the Netherlands. Eligible patients who receive an ARFID diagnosis in one of the centers, will be informed both orally and through a written information letter. After receiving the study information, they will be provided with the option to fill in their contact information via a QR code. Then, after a one-week consideration period, the researchers will contact the participant and send a link to a digital informed consent form. Participants will receive €10 for every assessment point (€40 in total).

### Inclusion/exclusion criteria

Participants must be 18 years or older, with ARFID as their primary diagnosis requiring clinical intervention, as determined during routine intake assessment by a licensed clinician at the participating treatment center. Exclusion criteria are: (1) having received more than four CBT sessions for ARFID in the previous year; (2) a current diagnosis of another eating disorder; (3) current use of enteral feeding; (4) history of psychosis; (5) severe psychological comorbidity requiring primary treatment (e.g., addiction, severe depression, suicidal ideation); (6) intellectual disability (given the cognitive demands of the treatment protocol); and (7) pregnancy. Medication is not an exclusion criterion but preferably remains stable (changes are registered). Underweight patients are included if outpatient treatment is deemed sufficient.

### Study design

**Fig 1:**
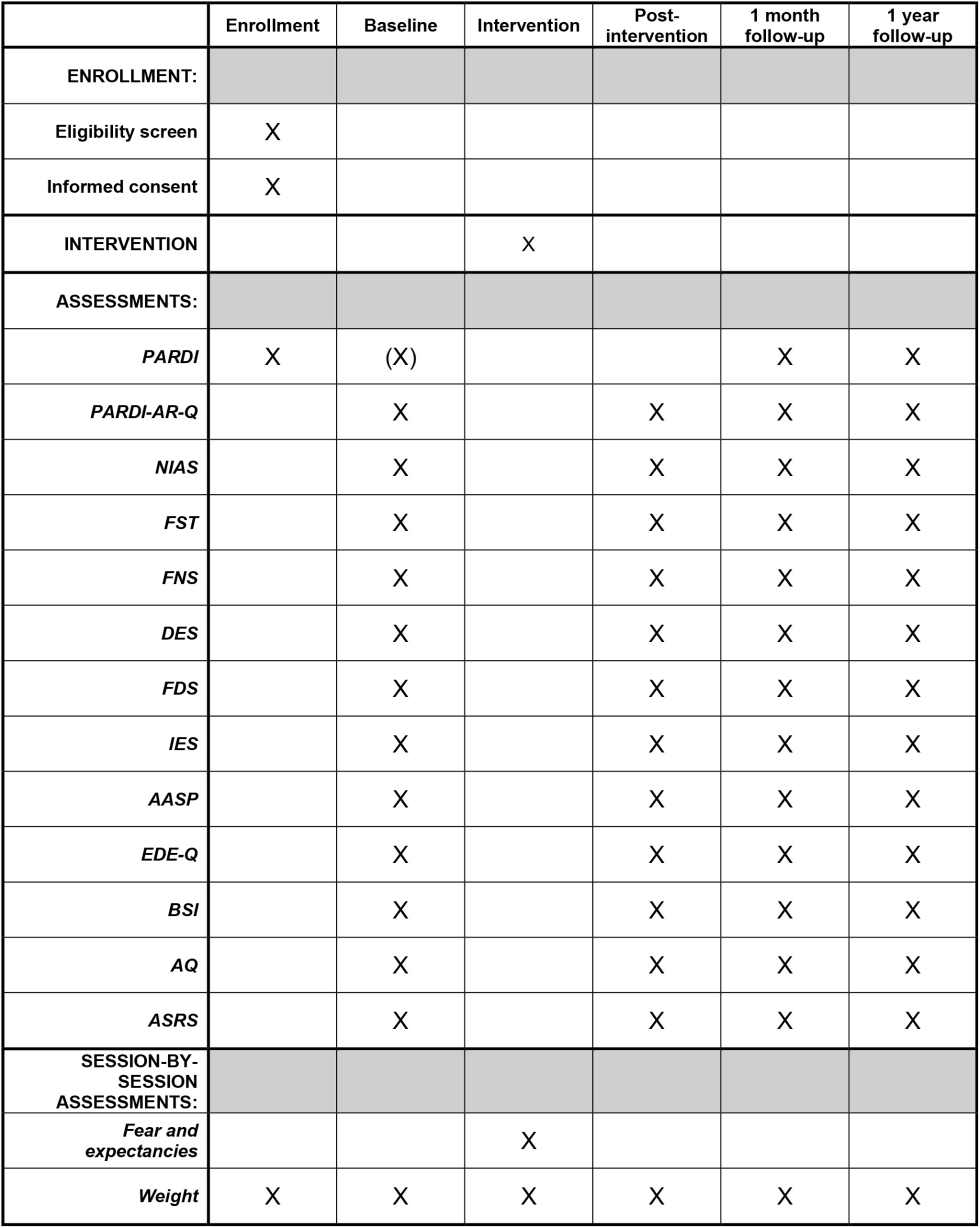
Participant timeline: Enrollment, intervention, and assessments.

Given the early stage of the treatment development for ARFID, and the specific research questions on feasibility, ARFID profiles, and comorbidity, the current study uses a prospective, uncontrolled, repeated measures design. Participants will complete questionnaires and a semi-structured interview before and after treatment and at 1-month and 12-month follow-up points. The treatment consists of 25 weekly, 1-hour sessions. Questionnaires will be filled out online (via Qualtrics). Beside the four assessment points, specific measurements regarding fear and expectancies will also be recorded during each exposure session. Fig 1 shows a summary of the timeline and assessment points for participants, in line with the Standard Protocol Items: Recommendations for Interventional Trials (SPIRIT) guidelines [27].

### Measures

**Pica, ARFID, and Rumination Disorder Interview (PARDI):** The key primary outcome is the severity score derived from the PARDI [28,29]. The PARDI is a clinician-administered, semi-structured interview developed to systematically assess the presence and severity of pica, ARFID and rumination disorder. It contains 100 questions, of which the majority is scored on a 7-point Likert scale (0–6). An overall ARFID severity score (0–6) can be calculated based on 17 items, providing a dimensional index of daily burden. Furthermore, the PARDI generates three profile scores, corresponding with the three ARFID profiles (Sensory sensitivity, Lack of interest, and Fear of aversive consequences), allowing for a more nuanced understanding of the symptoms. The profile scores will be used for additional analyses on the effectiveness across the different ARFID profiles. The PARDI has demonstrated adequate to good reliability and evidence for construct and criterion validity [28,30], although these findings are based on younger age groups (9–23 years).

**Weight:** Secondly, we will assess treatment outcome by weight gain, specifically for participants who are underweight (BMI < 18,5 kg/m^2^) before treatment. Participants will be weighed during the intake phase at the treatment center, during the first treatment session, and subsequently either every fifth session (participants with normal weight or overweight) or each session (underweight participants). At the one-month follow-up, participants will be weighed by the therapist and self-report their weight. At the one-year follow-up, they will only self-report, given that they are not in treatment anymore and since all follow-up measurements will be conducted online.

**Fear and expectancies:** To investigate our fourth research question, participants rate level of fear and endorsement of dysfunctional cognitions before and after every exposure. For this, we use an unmarked 100 mm visual-analogue scale (VAS). Fear levels will be assessed in relation to the specific product or situation practiced before and after each exposure session. In contrast, endorsement of dysfunctional cognitions will be assessed before and after each exposure session using the same predetermined set of cognitions (the so-called ‘catastrophic causal misinterpretations’ or CCM’s) across sessions. Furthermore, for each exposure, participants will also rate the credibility of a CCM linked to that specific exposure, both before and after this exposure (similar to the OptExNexus in [31]; see ‘Intervention’). This approach enables examination of within- and between-session changes in fear and cognition.

**Pica, ARFID and Rumination Disorder Interview ARFID Questionnaire (PARDI-AR-Q):** The PARDI-AR-Q [32,33] is a questionnaire based on the PARDI, focused solely on ARFID. It consists of 32 items, including items questioning the DSM-5 criteria but also the different ARFID profiles. Like the interview, the questionnaire generates a general severity score and three profile scores, all ranging from 0 to 6. The PARDI-AR-Q has shown good to excellent internal consistency for all three subscales and evidence of convergent and discriminant validity [32].

The first PARDI is conducted by a clinician in the treatment center, either during the intake phase or right before treatment. If the PARDI was conducted more than 3 months before the treatment, e.g., due to a long waitlist, it is administered again. At follow-up times, the PARDI is conducted by the researchers.

**Nine Item ARFID Screen (NIAS):** The NIAS [34,35] is a brief nine-item self-report questionnaire used to screen for ARFID in adolescents and adults. The items – 3 per ARFID profile – are rated on a 6-point Likert scale, ranging from 0 (“completely disagree”) to 5 (“completely agree”), resulting in a total score (0–45) and 3 profile scores (0–15). The NIAS does not include items assessing DSM-5 criteria for ARFID or other eating disorders. The instrument has been validated in a large community sample [34] and showed high internal consistency within the subscales, good test-retest reliability, and evidence for convergent and discriminant validity.

**Food Selectivity Test (FST):** The FST (described in [23]) is a list of 145 food items that the participant rates with either red (“I would not/cannot eat it”), green (“I would/can eat it”), orange (“I would/can eat it, if…”, referring to certain conditions such as a specific brand or accompaniment), or yellow (“I have never eaten it”). From this, we will calculate the food acceptance score (FAS), defined as the proportion of “green” items relative to all other products. An increase of FAS over time indicates an increase of accepted food products. The FST has not yet been validated.

**Food Neophobia Scale (FNS):** The FNS [36] is a self-report questionnaire that measures reluctance to eat and/or avoidance of novel foods. The 10 items are scored on a 7-point Likert scale, ranging from 1 (totally disagree) to 7 (totally agree). The sum of the items (after reversing items 1, 4, 6, 9, and 10) results in a total score ranging from 10 to 70. The English version has a satisfactory test-retest reliability and internal consistency [36].

**Disgust:** We will use two questionnaires to measure disgust sensitivity: one general disgust questionnaire and one disgust questionnaire that is specific to food.

***Disgust Emotion Scale (DES):*** The DES [37] is a 30-item questionnaire assessing disgust sensitivity across five domains: (1) rotting foods, (2) blood and injections, (3) smells, (4) mutilation and death, and (5) small animals such as insects, rodents, or mollusks. The DES is scored on a 5-point Likert scale, ranging from 0 (no disgust at all) to 4 (extreme disgust). Olatunji et al. [38] validated the DES in both U.S. and Dutch student samples, demonstrating good internal consistency for the total scale, adequate consistency for the subscales, and evidence of convergent validity.

***Food Disgust Scale (FDS):*** The FDS [39] is focused solely on disgust elicited by food. We translated the short version [40] from English [41] and German [39] to Dutch. Afterwards, the Dutch version was back-translated by a native English and a native German speaker and slightly adjusted based on this. The short version contains eight items, with one item per category: animal flesh, poor hygiene, human contamination, mold, decaying fruit, fish, decaying vegetables, and living contaminants. The scale is scored on a 6-point Likert scale ranging from 1 (not disgusting at all) to 6 (extremely disgusting). The original short version of the FDS has demonstrated good model fit, acceptable internal consistency, good test-retest reliability, and evidence of convergent and incremental validity [39]. A cross-cultural study further showed that the short FDS had an adequate-to-good model fit in nine of the ten countries examined and good construct and criterion validity [40]. The Dutch version has not yet been validated.

**Impact of Events Scale (IES):** Because ARFID can be preceded by a traumatic experience (e.g., related to food or the oral region), we also assess two PTSD dimensions, namely intrusions and avoidance. For this, we will use the Dutch version of the IES [42,43]. This version consists of 15 items, scored on a 4-point Likert scale ranging from “not at all” to “often”. The total score ranges from 0 to 75, with higher scores indicating a stronger reaction to the traumatic experience. The Dutch version of the IES has been validated by Van der Ploeg et al. [44] and demonstrated good psychometric properties.

**Adolescent/Adult Sensory Profile (AASP):** To assess how an individual processes and responds to sensory stimuli, we will administer the AASP [45]. This self-report questionnaire consists of 60 items, covering 6 sensory processing categories: Taste/Smell, Movement (vestibular/proprioceptive), Visual, Touch, Activity Level, and Auditory. All items are divided into four quadrants, namely Low Registration, Sensation Seeking, Sensory Sensitivity, and Sensation Avoiding, reflecting neurological thresholds and self-regulation strategies. Scores on these quadrants range from 15 to 75. The Dutch version shows adequate internal consistency [45].

**Eating Disorder Examination-Questionnaire (EDE-Q):** Although a concurrent eating disorder is an exclusion criterion, we will also administer the EDE-Q [46] because some (subclinical) symptoms might go unnoticed or might not be reported to the clinicians. The EDE-Q is a self-report questionnaire consisting of 28 items, scored on a 7-point Likert scale (0–6). It includes four subscales: Restraint, Eating Concerns, Weight Concerns, and Shape Concerns. Aside from these, a total score can be calculated based on the average of the four subscale scores. The EDE-Q has demonstrated good reliability and validity [47–49]. The Dutch version also shows good internal consistency and discriminant validity [50].

**Comorbid psychiatric and neurodevelopmental disorders:** To assess potential comorbid psychiatric and neurodevelopmental disorders, information will be obtained from both the participant and the clinician. In addition, the following questionnaires will be administered at all four assessment points to gather further information on potential symptoms that were not reported.

***Brief Symptom Inventory (BSI):*** The BSI [51] is a 53-item self-report questionnaire that measures psychological symptoms and distress during the past seven days. Items are rated on a 5-point Likert scale ranging from 0 (not at all) to 4 (extremely). The BSI covers nine symptom dimensions: Somatization, Obsession-Compulsion, Interpersonal Sensitivity, Depression, Anxiety, Hostility, Phobic Anxiety, Paranoid Ideation, and Psychoticism. In addition to the subscale scores, we calculate the Global Severity Index (GSI), which reflects overall psychopathological distress and is derived from the mean score across all items. The BSI has been thoroughly validated, showing high internal consistency, good test-retest reliability, and good convergent and construct validity [51].

***Autism-Spectrum Quotient (AQ):*** To assess autistic traits, participants will complete the AQ [52]. The AQ consists of 50 items, rated on a 4-point Likert scale ranging from 1 (completely agree) to 4 (completely disagree). It has shown good internal consistency and test-retest reliability [53].

***Adult ADHD Self-Report Scale (ASRS):*** We will use the short version of the ASRS [54] to assess ADHD symptomatology. This version consists of 6 items, based on DSM-IV [55] criteria for ADHD. Each question asks how often a symptom occurred over the past 6 months on a 5-point Likert scale ranging from 0 (never) to 4 (very often). The 6-item ASRS has shown acceptable internal consistency and test–retest reliability, and good concurrent (criterion) validity with clinician diagnoses [56].

### Treatment

A treatment manual was developed, based on the CBT protocol of Dumont and colleagues [23]. The manual was adapted for implementation within a secondary care, outpatient setting, and informed by consultations with some of the treatment centers regarding number and duration of sessions.

The treatment consists of 25 weekly 1-hour sessions. It starts with 5 preparatory sessions, covering the rationale, treatment goals, identifying 3–4 CCM’s, avoidance and safety behavior, a case formulation of the current behavior, and making a list of exposure exercises. Furthermore, patients will fill out a food diary (one week in the beginning of treatment and at the end of treatment) and the FST.

After this, the treatment continues with 20 exposure sessions. The exposure is based on the inhibitory learning approach, as described by Craske and colleagues [24,31,57]. During the sessions, patients will fill out exposure forms similar to the OptExNexus inhibitory exposure log [31]. The therapists are instructed to refrain from explicit cognitive restructuring to optimize expectancy violation during exposure. Aside from exposure, there is also room for 1–3 behavioral experiments. During the last 3–5 sessions, the therapist and patient formulate a relapse prevention plan, and during exposure sessions 10, 15 and 20, the therapist evaluates the treatment (e.g., therapeutic alliance, progress, possible problems) with the patient. Throughout the treatment, patients fill out homework forms to log how often and what they practice at home.

Because the participants are adults, the protocol does not systematically involve parents. However, it is possible to involve close family or partners if necessary. Consultations with a dietitian are limited to a maximum of four during treatment, to examine the effects of CBT as purely as possible without concurrent interventions. However, a dietician can assist in identifying potential nutritional deficiencies and in determining priorities for the introduction of new foods. The number of online sessions is also limited to five in total.

Across the 11 treatment centers, 55 therapists were trained in the protocol. They were required to have experience with ARFID and CBT. The therapists were trained by the researchers (MM and SM), one of whom is a certified cognitive-behavioral therapist and supervisor (SM), during a four-hour online training. Before this training session, they read the treatment manual and watched two roleplaying videos, one of an exposure exercise and one of a behavioral experiment. During the training, they practiced with executing exposure in accordance with the inhibitory model.

To assess treatment fidelity, therapists fill in a checklist each session and report what components or CBT techniques were covered. They also report the time of the separate components and any remarks or additional information. Additionally, to avoid therapist drift, researchers meet monthly with the therapists for supervision in small groups.

### Data analysis

Primary analyses will examine changes in ARFID severity over time (research question 1). To account for the three-level data structure, where observations are nested within participants and participants are nested within centers, a linear mixed-effects regression model will be used. A starting model includes Time (pre-treatment, post-treatment, 1-month follow-up, 12-month follow-up) as a categorical fixed effect. In addition, three ARFID profile scores along with their interactions with Time will be entered as continuous predictors (research question 2). Participants and treatment centers will be included as a random effect. Finally, an unstructured covariance matrix will be specified and simplified if supported by the data. With this model, a main effect of time will test whether PARDI severity decreases during treatment, and a Time x Profile interaction will test whether trajectories differ across the three profiles. Post-hoc comparisons will be corrected for multiple testing. Explorative analyses will assess whether specific comorbidities (e.g., ASD, ADHD, anxiety disorders) predict treatment outcomes (research question 3). This will be done by including the comorbidities separately in the chosen mixed model that best describes the trend in the severity scores over the different timepoints. These will be included separately rather than simultaneously, to avoid overfitting of our model. Finally, correlations between decrease in PARDI severity scores and, respectively, fear reduction scores and expectancy violation scores will be examined (research question 4).

### Sample size

The starting model has 28 parameters for fixed effects, random effects, and the covariance structure. We aim to have approximately 10–15 independent observations per parameter, requiring 280–420 independent observations in total. Because four observations from the same participant are correlated, the number of independent observations per participant is fewer than four. For small to moderate within-person correlations, we can count only two to three independent observations per participant, meaning we need 90–210 participants. Note that estimating interaction terms often requires more observations and that missing values are common in longitudinal research; therefore, we aim to recruit 120 participants. Recruitment feasibility is further constrained by the relatively low prevalence of ARFID in general population samples. ARFID intervention literature to date frequently reports small to moderate sample sizes [58]. This justifies the intended sample size.

### Ethical and safety considerations

This study has been approved by the Ethics Review Committee Psychology and Neuroscience (ERCPN) in Maastricht, the Netherlands (ERCPN-276_07_01_2024) in March 2024. In May 2024, the Medical Ethics Review Committee azM/UM has decided that the Medical Research Involving Human Subjects Act (WMO) does not apply to this study, and thus does not need an additional approval. The study has been preregistered in AsPredicted (#212,212) in February 2025. The trial was registered in the Overview of Medical Research in the Netherlands (NL-OMON6121) in May 2026.

The protocol of this study, as approved by the ERCPN, is attached as a supporting information file (S2 File) and adheres to the guidelines outlined in the SPIRIT checklist for trial protocols (S1 File).

The study poses no significant risks to participants. Although exposure-based therapy inherently involves some degree of discomfort, this treatment is already routinely delivered across multiple clinical centers. Any discomfort arising during treatment is addressed by the clinician. Participants may withdraw from the study at any time without consequence for the treatment. As in standard clinical practice, treatment may be modified or discontinued if indicated, as determined by the treating clinician.

### Dissemination

The results of this study will be published in peer-reviewed journals and presented at national and international conferences. Results will also be shared with the participating clinical centers and disseminated through relevant stakeholders to facilitate translation into clinical practice.

### Data management

Participants will be assigned a participant number. Pseudonymized data will be stored on a dedicated research data management (RDM) server, that only the main researchers can access. The key linking participant numbers to identities will be stored in a separately secured folder on the same server. Data will be retained for ten years following the last publication, in accordance with ERCPN guidelines.

### Status of the study

Participant inclusion initiated on January 21, 2025 and will continue until July 1, 2026. Post-treatment assessments are expected to be completed by January 2027, followed by the 1- and 12-months follow-up assessments. Data collection will be complete in July 2027. Results are expected to be reported in the second half of 2027.

## Discussion

In this multicenter prospective study, we investigate the effectiveness and mechanisms of an exposure-based CBT program for adults with ARFID. Given the limited availability of evidence-based interventions for adults, this study addresses a substantial gap in the literature. The design not only allows for the evaluation of overall treatment effects but also examines whether changes vary across the three ARFID profiles. The inclusion of session-level measures of fear reduction and expectancy violation provides an opportunity to examine mechanisms of change, an aspect that has not yet been researched in the field of ARFID.

One of the strengths of this study is its ecological validity. Treatment is delivered in routine clinical practice across multiple centers, making the findings more generalizable to real-world settings. Longitudinal assessment, including a 12-month follow-up, further strengthens the study by enabling evaluation of sustained treatment effects. Another strength is the use of gold-standard clinician-administered assessment (the PARDI), complemented by multiple secondary outcome measures to capture improvement.

At the same time, several limitations should be acknowledged. Treatment integrity will be monitored through checklists and supervision meetings, but sessions will not be audio- or video-recorded, which limits the ability to assess adherence independently. Therapists vary in experience, introducing additional heterogeneity in treatment delivery. Although this variability may reduce internal validity, it enhances ecological validity by reflecting typical clinical practice. The absence of a control group restricts causal inference; however, this naturalistic design enables the inclusion of a broader and more representative clinical population. Lastly, the exploratory analyses concerning comorbidities and additional clinical variables should also be interpreted cautiously due to the risk of overfitting and multiple comparisons.

Despite these challenges, this study is expected to make a meaningful contribution to the ARFID literature. It will provide data on the effectiveness of a structured CBT protocol for adults, clarify whether the three ARFID profiles respond similarly to treatment, and offer insights into exposure-related mechanisms of change. Findings may inform clinical practice, guide refinement of the treatment, and serve as a foundation for future controlled trials.

## Supporting information

S1 File SPIRIT 2025 checklist. (PDF)

S2 File Protocol as accepted by the Ethics Review Committee Psychology and Neuroscience (ERCPN) – English translation. (PDF)
