# Supplementary material for: Cognitive-behavioral therapy focused on inhibitory learning, for adults with avoidant/restrictive food intake disorder (ARFID): Study protocol of a prospective study
Source: PLoS One. 2026 Jul 31;21(7):e0354232. doi: 10.1371/journal.pone.0354232 (PMC13426971; doi:10.1371/journal.pone.0354232)
Supplement: S2 File — (PDF) [file pone.0354232.s002.pdf]

Original protocol as accepted by the ethics committee

Question #	Question	Answer
1a	Applicant name	Malou Masereel
1b	Email	malou.masereel@maastrichtuniversity.nl
1c	Department	CPS
1d	Correspondence language	Dutch
2	Study name	NL: Never too old to learn? Cognitive behavioral therapy focused on expectancy learning, for (young) adults with avoidant/restrictive food intake disorder (ARFID).
3	Study type	Regular Protocol
4	Organisation where research is carried out	PhD project within FPN (CPS). Participating centers: PsyQ (The Hague/Rotterdam), Co-Eur (Maastricht, Hoensbroek, Roermond, Vught, Utrecht), SeysCentra (Maastricht, Malden, Utrecht, Zwijndrecht), ARFID outpatient clinic of MUMC+. Novarum (Amsterdam) and Emergis (Goes) have expressed intention to participate. Funded by 'Stichting tot steun VCVGZ'.
5	Relevant earlier ERCPN-approved protocol(s)	164_16_03_2016: Dumont et al. (2019): A new CBT for adolescents with ARFID in a day treatment setting (proof-of-concept, n=11). Protocol adapted for adults in outpatient, secondary care, individual format.<br>Master_189_20_03_2018: Waiting list study for a master's thesis via SeysCentra, using many of the same instruments (Food Selectivity Test, Food Neophobia Scale, food diary, VAS, CCMs).
6	Background of the study	ARFID is an eating disorder involving insufficient and/or highly selective eating, associated with physical and/or psychosocial problems (~1% of adults). Can co-occur with anxiety disorders, mood disorders, ASD, or ADHD. DSM-5 describes three profiles: (1) lack of interest in food/eating; (2) sensory avoidance; (3) fear of aversive consequences. A CBT protocol for adolescents based on inhibitory learning principles showed promising pilot results (n=11), but research for adults in outpatient, individual care is lacking.
7	Hypotheses	1) CBT based on inhibitory learning is effective for adults with ARFID (expected: significant reduction in ARFID scores over time). 2) The program works across all three ARFID profiles (expected: no interaction effect between time and profile). 3) [Exploratory] Do comorbid disorders (e.g., ASD, ADHD, anxiety disorders, PTSD, OCD) affect the treatment outcome? 4) Does the program work via expectancy disconfirmation (inhibitory learning) or anxiety reduction/habituation (expected: better treatment outcomes correlate more with reduced fearful expectancies).
8	Why this study	As ARFID is a relatively new diagnosis, no evidence-based treatment is currently available for adults with ARFID.

		Furthermore, no evidence-based outpatient (secondary care) treatment currently exists, although an intensive day treatment program (four weeks) for adolescents with severe ARFID, based on the same principles, has already demonstrated promising results. There is a substantial demand for such programs, as ARFID can have considerable consequences for the physical health and/or psychosocial wellbeing of affected individuals. The ARFID outpatient clinic at MUMC+ has been offering this program to adults for 2.5 years, and PsyQ has also been trained in this approach by our team (i.e., SeysCentra; Mulkens and colleagues), yet there remains a need for standardized research into this treatment.
9	Required actions by participants	Treatment of ARFID in (young) adults is already being provided in the participating centers, based on CBT. However, in order to address the research questions, all centers will standardize their diagnostic and treatment procedures (following a training session) and assessments will be conducted in a structured manner at identical timepoints across centers. Following intake and diagnostic assessment, including determination of overall ARFID severity and scores on the three ARFID profiles using the Pica, ARFID, and Rumination Disorder Interview (PARDI) and assessment of potential comorbidities (ASD, ADHD, anxiety disorder, OCD, PTSD), the CBT treatment program will be offered (3 pre-sessions and 20 weekly sessions).<br>Assessments will take place before and after treatment and at 1- and 12-month follow-up. Between the second and third assessment points, so after treatment, a one-month treatment pause will occur. After the third assessment, it will be evaluated whether further treatment is indicated. Any additional treatment sessions received by participants will be recorded.<br>At each assessment point, participants will complete a food diary for one week (evaluated for nutritional deficiencies by a dietitian), and height and weight will be recorded (self-reported by participants via Qualtrics at follow-up, and recorded by the treating clinician before and after treatment; participants provide consent for access to these data). In addition, the following questionnaires and interviews will be administered at each assessment point: the PARDI-AR-Q (PARDI-ARFID Questionnaire), PARDI interview, NIAS (Nine Item ARFID Screen), FNS (Food Neophobia Scale), EDE-Q (Eating Disorder Examination Questionnaire; to screen for other eating disorders), food selectivity test, DES (Disgust Emotion Scale; to assess disgust sensitivity), IES (Impact of Event Scale; to assess traumatic experiences), Sensory Profile (to assess sensory sensitivity), AQ (Autism Quotient;

		to assess autistic traits), and BSI (Brief Symptom Inventory; to assess general psychopathology).<br>During the weekly therapy sessions, participants will complete visual analogue scales (VAS) regarding anxiety levels and expectations (Catastrophic Causal Misinterpretations, or CCMs) in relation to the food being practiced (before and after exposures), and structured exposure forms will be used to inventory and measure these expectations.
10	Use of equipment	None
11	Independent variables	Independent variables: time point (pre, post, 1-month follow-up, 12-month follow-up), ARFID profile (lack of interest, sensory sensitivity, fear of aversive consequences), comorbidity (ASD, ADHD, anxiety disorder, PTSD, OCD). Primary dependent variables: ARFID severity scores (PARDI), anxiety levels, expectancies (CCMs). Secondary dependent variables: weight/height, food selectivity test acceptance score.
12	Statistical approach	Prospective study without randomization. Statistics will be carried out in SPSS. We will conduct repeated measures ANOVAs for research questions 1-3 and linear regression for research question 4.
13	Number of participants	Power analyses for the treatment study were conducted with GPower 3.1. Sample size was calculated based on 'intention-to-treat'. It was calculated that a sample size of 30 patients per profile would be needed (N=90) for a power of 80% with two-sided testing ($p < 0.05$) to detect a difference in global PARDI score between the groups and between pre- and post-measurement. The total number of patients that can be invited in three years is estimated at 214 (without Novarum). For the study N=90 participants ('completers') are needed, with recruitment up to N=120 to ensure sufficient participants per profile. With 214 potential candidates of whom approximately 30-45% will refuse participation OR drop out earlier (based on historical experience), we guarantee being able to include 118-150 patients. That is already sufficient for the current research questions. Should Novarum ultimately also participate, this number will increase further to 200-250.
14	Maximum participant load	Participants will complete the CBT treatment programme of 3 pre-sessions and 20 weekly sessions. Before (during the intake) and after treatment and at 1 and 12 months follow-up, measurement moments take place of approximately 1.5 hours. These measurement moments consist of online questionnaires (Qualtrics) and an online interview (PARDI). The interview is conducted by the researcher. If the PARDI was already administered during the intake and treatment is

		started within three months after its administration, this data is used for the pre-measurement. We ask participants for consent for use of these data, as well as other data from the intake (height, weight, comorbidity).
15	Participant type	Patients. Other: adult (18+) patients with ARFID (who meet inclusion criteria) from the centres PsyQ The Hague/Rotterdam, Co-Eur (all 4 locations), SeysCentra (all 4 locations), MUMC and possibly Novarum Amsterdam and Emergis (Goes).
16	How will testing take place	Other: Intake and treatment sessions take place face-to-face ('in person'). Questionnaires are administered online via Qualtrics. The PARDI interview is administered online via video call.
17	Recruitment and anti-bot measures	Participants are recruited via the participating treatment centers when they register with complaints fitting ARFID. After the intake procedure (separate from our study), the centers assess whether they qualify for the study. If this is the case, information about the study is provided during the advice consultation based on the information letter (this is also verbally explained by the intake worker of the relevant center). A reflection period of one week is given, after which patients can sign the informed consent.
18	Does the study involve deception?	No
19	Involves video or audio recording of participants?	Yes
20	How are the recordings made, handled and stored?	The ERCPN procedure for making audio recordings is followed with regard to handling/storing of the recordings, informed consent etc. The audio recordings are made by the therapists in the respective centres. They must then immediately send the recordings digitally via SurfFileSender after recording and subsequently erase the memory card, as discussed with FPN Privacy. A password for the encryption is agreed with the therapists via a separate channel. No names of participants are mentioned on the audio recordings, only participant number, date and a code to identify the therapist. The audio recordings are stored in accordance with the ERCPN procedure in the RDM privacy folder. A list with the participant numbers and corresponding names is kept in a separate file from this, but also in the privacy folder. The audio recordings are retained for 10 years after the last publication, so that 'treatment integrity' can be assessed at later moments if necessary.
21	What acts or situations will be recorded?	The therapy sessions will be recorded on audio (voice recorder) in order to assess the quality of the

		treatment/adherence to the protocol on a random basis. The recordings will also be used for intervision.
22	Additional info	We are awaiting the declaration of local feasibility from PsyQ and MUMC (they may first want a non-WMO declaration from the METC). Novarum is still considering internally whether participation is possible and there may also be an additional centre participating (Emergis, affiliated with PsyQ via Eetstoornis Experts Netwerk or EEN), which may mean it takes some time before we have received all letters. The declarations of local feasibility from Co-Eur and SeysCentra have been added as an appendix. Regarding data sharing, we have been in contact with Privacy FPN to draw up a data transfer agreement. However, they indicated that it is very busy and the processing period can take three to six weeks.
23	Information Letter	Model
24	Declaration of Consent	Model 1 for non-anonymous research
25	Debriefing Letter	Model
26	Consent given actively	Yes, provided on paper
27	Inclusion/Exclusion criteria	Yes. Exclusion criteria are: age <18 years, IQ < 70; more than 4 sessions CBT for ARFID in the past year; diagnosis of another eating disorder than ARFID; history of psychosis; currently receiving tube feeding; severe comorbid psychiatric problems requiring primary care (such as addiction, severe depression or suicidality) and pregnancy. Medication is not an exclusion criterion but the use of psychotropic medication is registered and should preferably be kept constant during the duration of the study, so that the influence of the medication remains constant.
28	Risks of discomfort	Yes: There are no risks or discomforts associated with the study. Therapies for ARFID will by definition cause some discomfort (due to the exposure), but these treatments are already being provided in various centres. The questionnaires are also already administered in practice. If discomforts arise in relation to the treatment, this is also addressed during the therapy.
29	Reward for participants	Yes: Participants receive a voucher of €10 per measurement moment (total of 4 if participants complete the full study).
30	Type of data collected	Personal: name, date of birth, address, BSN number (for voucher), audio recordings of the sessions without name of participant but with participant number, a code to identify the therapist and the date. Physical characteristics: height and weight. Demographic: age,

		gender. Health-related data: questionnaires and interview re. psychopathology and psychology, food diary...
31	Individual feedback	Yes: If the data are part of the therapy or standard procedure of the intake, participants receive feedback from the therapist as usual (e.g. they receive feedback on the questionnaires they complete as usual during the intake).
32	Advertisement	Model
33	Estimated date data becomes static	October 2027
34	Possible to save data immediately in secure storage	No: There is certain data that is collected by the therapists of the various centers. This must first be sent via SurfFileSender to the responsible researcher. This happens as soon as possible after obtaining the data. The researcher then immediately places the data on the RDM server and the data on SurfFileSender is deleted.
35	Access to anonymous data	Malou Masereel (PhD candidate), Sandra Mulkens (supervisor)
36	Access to personal data	Malou Masereel (PhD candidate), Sandra Mulkens (supervisor)
Checklist	Information Letter	✓ Check
Checklist	Advertisement	✓ Check
Checklist	Debriefing	✓ Check
37	Information Letter	fpn_ercpn_information_letter_NL_2024-02-28.docx
38	Declaration of Consent	fpn_ercpn_informed_consent_NL.doc
39	Debriefing	fpn_ercpn_debriefing_letter.docx
40	Advertisement	fpn_ercpn_advertisement.docx
41-43	Letter of Permission / Confidentiality / Reward Form	(not uploaded)
44	Other	fpn_verklaring lokale uitvoerbaarheid_SeysCentra.pdf
45	Other	fpn_Verklaring lokale uitvoerbaarheid_Co-Eur.docx
46-48	Other	(not uploaded)

Amendments – accepted by ethics committee

Amendment 1

- Due to practical constraints, we will not make audio recordings.
- We have increased the number of sessions in the protocol from 23 (3 + 20) to 25 (5 + 20), because the treatment centers considered 3 preparatory sessions too little.
- After consultation with [a member of the ethics committee], we adjusted the procedure for the informed consent to a digital method. When a patient is eligible for the study, the clinician of the treatment center gives information about the study during the last session of the intake procedure

(feedback session), using the information letter. If the patient is interested and agrees to be contacted by the researchers, they receive a QR code or link to a Qualtrics questionnaire with the question if they agree to be contacted and where they can provide their name, e-mail address, phone number, center and intake clinician.

- Because of these changes, we also adjusted the information letter accordingly.
- We will add the following questionnaires: short ADHD Self-Report Scale (ASRS, 6 items) in order to assess ADHD symptoms; and short Food Disgust Scale (FDS, 8 items), translated by us, in order to assess disgust in regards to food.

Amendment 2

We have made a flyer to put in waiting rooms, so that the study is visible for patients and clinicians. The inclusion/recruitment still happens exclusively via the clinicians in the intake procedure of the centers. They decide after the intake procedure if someone is eligible for the study and subsequently give information during the feedback session (both orally and via the information letter). The only purpose of the flyer is to make the study more visible for patients (in order to minimize the possibility of clinicians forgetting to give information about the study).

Amendment 3

- We added five extra treatment centers (to increase inclusion and promote geographic distribution), namely: GGZ Centraal, GGZ Oost-Brabant, Altrecht (Rintveld), Rivierduinen (Ursula) and GGZ Friesland.
- Due to this, we had to adjust the information letter and flyers

Amendment 4

- We decided to omit the PARDI interview immediately after treatment (2nd time point). This is because the questions of the interview assess symptoms over the past 1-3 months. If we were to administer the interview immediately after treatment and 1 month after treatment, these periods would overlap. Furthermore, the interview after treatment would be a reflection of not only the symptoms right after the treatment, but also of the preceding 3 months (and thus, during treatment). This is too broad of a time period to properly measure the symptoms after treatment. In contrast, the interview 1 month after treatment reflects a more meaningful period for evaluating symptom change.
- We adjusted the information letter accordingly. The sentence that we changed is:

  Op deze vier momenten zal van u worden gevraagd om online vragenlijsten in te vullen en bij de follow-up metingen (1 en 12 maanden na de behandeling) zullen we bij u een interview afnemen van 45-60 minuten (online).

  EN: *At these four time points, you will be asked to fill out online questionnaires and during the follow-up measurements (1 and 12 months after treatment), we will conduct an interview of 45-60 minutes (online).*
